# Health, hope and worry: A qualitative study of people with diabetes' experiences with anti‐VEGF treatment

**DOI:** 10.1111/dme.70334

**Published:** 2026-04-20

**Authors:** J. K. Leksell, E. Granstam, T. Granström, E. Nerpin

**Affiliations:** ^1^ Department of Medical Sciences, Clinical Diabetology and Metabolism Uppsala University, Akademiska Sjukhuset Uppsala Sweden; ^2^ Department of Surgical Sciences Uppsala University Uppsala Sweden; ^3^ School of Education, Health and Social Studies Dalarna University Falun Sweden; ^4^ Department of Medical Sciences, Respiratory Medicine, Allergy and Sleep Uppsala University Uppsala Sweden

**Keywords:** anti‐VEGF therapy, emotional response, qualitative research

## Abstract

**Aims:**

Diabetic macular edema (DME) is a leading cause of vision loss among individuals with diabetes. While anti‐VEGF therapy is a widely used and effective treatment, limited research has explored participants' lived experiences throughout the treatment process.

**Methods:**

This qualitative study summarizes findings from longitudinal interviews conducted at 4 and 12 months after initiating intravitreal anti‐VEGF therapy. The analysis focused on participants' emotional, physical and psychosocial responses to treatment.

**Results:**

An overarching theme, ‘health, hope, and worry’, captured the ambivalent experiences of participants. Two main categories were identified: ‘hope and anxiety during injection treatment’ and ‘uncertainty and variation in visual outcomes’. Participants described a spectrum of physical discomfort and emotional reactions, influenced by expectations, bodily sensitivity and clinical context. Trust in healthcare providers, consistent care and adequate information were crucial for emotional resilience. Perceived treatment effectiveness varied, with some experiencing significant visual improvement and regained independence, while others reported little or no change, leading to frustration and anxiety about future vision.

**Conclusions:**

Anti‐VEGF treatment for DME is experienced not only as a clinical intervention but as an emotionally and physically complex journey. Participants' navigate fluctuating levels of hope and worry, shaped by their bodily experiences, emotional responses and expectations of outcomes. These findings highlight the need for empathetic communication, consistent care and individualized support to promote adherence and mitigate emotional distress. The insights from this study are especially relevant for multidisciplinary diabetes care teams.

## Graphical abstract

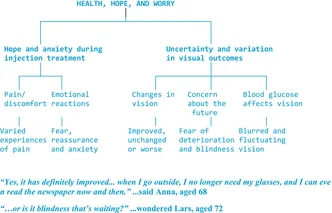

**What's new?**

Anti‐VEGF treatment for diabetic macular oedema is experienced as an ambivalent process characterised by health, hope, and worry, rather than a purely clinical intervention.

Persons’ experiences evolve over time, with adaptation to injections varying widely—some adjust, while others experience ongoing or increasing distress.

Perceived visual outcomes are highly variable and unpredictable, shaping emotional responses, expectations, and concerns about future vision.

Continuity of care, trust, and clear communication are essential to support coping, reduce distress, and promote treatment adherence.

## 1 INTRODUCTION

The fear of vision loss is among the most distressing complications of diabetes and is closely linked to diabetic macular edema (DME), a vision‐threatening consequence of diabetic retinopathy that can substantially impair daily functioning and quality of life.^1^ Anti‐vascular endothelial growth factor (anti‐VEGF) therapy is the current first‐line treatment for DME and has demonstrated efficacy in reducing macular oedema, improving visual acuity and enhancing vision‐related quality of life.^2^, ^3^, ^4^, ^5^, ^6^

Despite well‐established clinical effectiveness, understanding of how individuals experience anti‐VEGF treatment for DME remains limited. A recent scoping review of qualitative studies on intravitreal injections, which included our own pre‐treatment interview study, found that research has primarily focused on emotional and physical experiences of the injection procedure itself, highlighting themes such as fear of vision loss, anxiety about repeated injections, uncertainty regarding outcomes and discomfort due to the invasiveness of treatment.^7^ In our earlier pre‐treatment study, we identified two categories and an overall theme: ‘to be at a crossroads and a crucial phase in life with an uncertain outcome’, capturing both practical concerns about the treatment procedure and existential reflections, including hope for improved visual acuity and fear of deterioration.^8^

In our Swedish longitudinal study of individuals initiating anti‐VEGF treatment for DME, participants reported poor general health at baseline, alongside hopes for visual improvement and fears of disease progression.^8^, ^9^ Visual acuity and vision‐related quality of life improved during follow‐up, while general health showed little change, highlighting the need for qualitative insights on how treatment outcomes are perceived during the first year.^9^, ^10^, ^11^

Although qualitative research in this area is growing, most studies have been cross‐sectional and concentrated on the procedure rather than on how individuals perceive and interpret treatment outcomes over time. Consequently, knowledge is limited regarding how changes—or lack of change—in vision are interpreted, how expectations align with actual outcomes, and how treatment is integrated into daily life during the first year of treatment.

The aim of the present study was to explore how participants with DME describe and reflect upon their experiences, expectations and perceived impact of anti‐VEGF treatment during the first year, based on a combined analysis of qualitative interviews conducted at 4 and 12 months after treatment initiation.

## 2 METHODS

### 2.1 Research design and setting

Qualitative content analysis was used to identify patterns and changes in participants' narratives, with a focus on how their experiences, expectations and perceived impact of anti‐VEGF treatment developed during the first year.

### 2.2 Study sample

This interview study is based on the same cohort as previously described by Granström et al.^8^ Participants were recruited between May 2012 and February 2014 at two county hospitals in Sweden. The study included people with visual impairment due to DME who were about to undergo anti‐VEGF treatment and who met the inclusion criteria: age ≥18 years, no cognitive impairment and the ability to speak and understand the Swedish language without an interpreter. Participants were invited to participate in the interview study during a visit to the eye clinic and were included in the study until sufficient variation was achieved.

A total of 58 individuals were approached during visits to the eye clinic, of whom 21 agreed to participate in the interview study. One participant did not complete the 12‐month follow‐up interview. Inclusion continued until sufficient variation was achieved, as defined and assessed during recruitment in the original study.^8^ Sufficient variation was defined as variation in age, sex, type and duration of diabetes, visual ability, living situation, educational level and employment status. All participants completed interviews at baseline and at the 4‐month follow‐up. At the 12‐month follow‐up, one participant was lost to follow‐up; no additional participants were recruited for the follow‐up interviews. None of the participants had any prior relationship with the interviewer. Demographic characteristics, visual and clinical characteristics of the participants are presented in Table 1.

**TABLE 1:** Characteristics of the participants.

	Baseline (*n* = 21)	12 months follow‐up (*n* = 21^a^)
Female sex, *n* (%)	12 (57)	12 (57)
Age, mean (±SD)	69 (±11)	70 (±11)
Type of diabetes		
Type 1	1	
Type 2	20	
Diabetes duration, (year) range (median)	1–48 (17)	1–49 (17)
HbA_1c_, mmol/mol range (%) and [median] and (%)	44–84 (6.2–9.8) [66] (8.2)	46–87 (6.4–10.1) [66] (8.2)
Visual ability		
Normal	8	7
Mild visual impairment	9	12
Severe visual impairment	3	2
Change in visual ability at 12 months		
Improved		3
Unchanged		15
Deteriorated		3
Number of injections/treated eye, mean (range)		4.7 (1–7)
Number of participants with bilateral treatment		3
Number of visits		14 (9–17)
Living situation		
Living alone	10	
Cohabiting	11	
Education level		
Elementary school	11	
Middle school	6	
University	4	
Employment		
Retired	13	
Working	6	
Unemployed/sick leave	2	

*Note*: Data are given as n unless otherwise specified.

^a^ Baseline characteristics were available for 21 participants; 20 completed the 12‐month interview.

### 2.3 Data collection

Semi‐structured face‐to‐face interviews were conducted at the hospital 4 and 12 months after the initiation of anti‐VEGF treatment. The semi‐structured interview guide was developed by the research team, drawing on clinical experience and themes previously identified in pre‐treatment interviews with people living with DME.^8^ Two researchers (TG, JKL) drafted the initial guide, which was then refined through team discussions to ensure clarity and relevance. The guide focused on practical, emotional and bodily aspects of intravitreal anti‐VEGF treatment. No pilot interviews were conducted, but the first interview was reviewed for applicability and required no revisions. The 4 and 12‐month follow‐up guide was developed using the same iterative team‐based process.

Each interview lasted 30–40 min, was recorded and transcribed verbatim. The interview guide (Table 2) explored treatment experiences, emotional responses and the perceived impact of diabetes‐related visual impairment on daily life.

**TABLE 2:** Interviewguides after 4 and 12 months.

Second interview after 4 months:
*Interview questions*
What are your thoughts about the treatment you have received?
How did you experience receiving the three injections?
How did you experience the information you were given about the treatment?
Is anything missing? What was good to know?
How do you experience your visual acuity now compared to before the treatment?
Are you affected in your daily life by your vision impairment?
What would you say to someone who has been offered this treatment and is about to start it?
What are your thoughts about your diabetes right now? Has the awareness of vision problems affected your diabetes self‐care? In what way?
Third interview after 12 months:
*Interview questions*
How has it been since we last met?
How do you experience your visual acuity today?
How is your diabetes?
When did you realize that there was a connection between your diabetes and the change in your vision?
How do you view the future?
Follow‐up questions:
What do you mean?
How?
What?
Who?
Why?
Tell me more

### 2.4 Ethical considerations

The study was approved by the Uppsala Regional Ethical Review Board (ref: 211/264) and conducted per the Declaration of Helsinki. All participants gave written informed consent and were free to withdraw at any time without affecting their care.

### 2.5 Qualitative analysis

Conventional inductive qualitative content analysis was used. Based on qualitative theory, the interviews were analysed using content analysis as described by Graneheim and Lundman.^12^ An inductive approach allowed categories and themes to emerge from the data rather than forcing them into predefined models, in line with guidance from Graneheim et al.^13^ This was essential for capturing the complexity and variation in participants' evolving treatment experiences over time. Interviews were conducted and analysed sequentially. Saturation—defined as the point at which no new information or codes emerged—was reached after the 21st interview, when all themes began to recur. The sample size is consistent with empirical evidence indicating that thematic saturation is typically achieved within 9–17 interviews,^14^ thereby supporting the adequacy of our dataset. As the study followed the same individuals over time, adding new participants at the 4‐ and 12‐month follow‐ups was not compatible with the longitudinal design. Two researchers (EN and J.K. Leksell) coded independently and compared their coding; any differences were resolved through discussion until consensus was reached. No statistical inter‐rater reliability measures were applied, in line with qualitative analytic principles.

The analysis included the following steps:

- The transcribed interviews were read several times to achieve a thorough familiarity with the content and to gain an overall understanding of the data.
- Meaning units were identified within the text, then condensed and coded with labels that remained close to the participants' expressions (Table 3).
- The codes were compared and discussed in terms of similarities and differences to ensure they accurately reflected the underlying meaning units. The codes were then organized into subcategories, from which two main categories were developed.
- The coding process and the formation of subcategories and main categories were rigorously discussed to enhance credibility and consistency in the analysis.

**TABLE 3:** Examples from the content analysis.

Meaning unit	Condensed meaning unit	Code	Subcategory	Category
The first treatment was painless. The second was very painful and unpleasant, leaving an irritating black spot in the eye for over a week. The third felt like a large needle being pushed in and was extremely painful.	The first treatment was painless. The following ones were very painful, especially the last. An irritating black spot remained in the eye for over a week.	Pain and unpleasant treatments, followed by an irritating black spot in the eye.	Participants' experiences of pain and physical symptoms during treatment	Hope and anxiety—undergoing injection treatment
No, but it's okay… it's manageable… what worries me is the possibility of going blind, and then I'm also afraid of having a heart attack.	Worries about becoming blind.	Worries about vision impairment.	Emotional reactions and concerns	Hope and anxiety—undergoing injection treatment
Yes, it has definitely improved… when I go outside, I no longer need my glasses, and I can even read the newspaper now and then.	Vision has improved—no need for glasses outside and can sometimes read the newspaper.	Vision has improved.	Participants' experiences of how anti‐VEGF treatment affected their vision	Uncertainty and variation in visual outcomes
…the only thing one keep thinking about is really… how slow the process might be… if you… so to speak… can avoid any major problems until you die…	Continual wondering about how slow the process will be and whether major problems can be avoided.	Vague concern about future development.	Uncertainty regarding the long‐term effects of treatment on vision	Uncertainty and variation in visual outcomes
Yes, when my blood sugar is high, I feel like everything looks blurry… that's how it is, and it's not… it's not steady, so to speak, it goes up and down all the time.	High blood sugar seems to cause blurry and fluctuating vision.	Vision is blurry and unsteady, related to high blood sugar.	The perceived impact of blood glucose levels on vision	Uncertainty and variation in visual outcomes

## 3 RESULTS

### 3.1 Health, hope and worry: Experiences of anti‐VEGF treatment

The overall theme of *health, hope and worry* included two categories: *hope and anxiety—undergoing injection treatment* and *uncertainty and variation in visual outcomes*. These categories reflected the participants' mixed emotional and physical experiences during treatment, as well as the unpredictable effects on vision and concerns about long‐term outcomes (Table 4).

**TABLE 4:** Categories and subcategories in the qualitative results.

Main Category	Subcategory
Hope and anxiety—undergoing injection treatment	Participants experiences of pain and physical symptoms
	Emotional reactions and concerns
Uncertainty and variation in visual outcomes	Participants experiences of how anti‐VEGF treatment affected their vision
	Uncertainty regarding the long‐term effects of treatment on vision
	The perceived impact of blood glucose levels on vision

### 3.2 Hope and anxiety—Undergoing injection treatment

The first category was comprised of two subcategories: *participants' experiences of pain and physical symptoms during treatment*, and *emotional reactions and concerns*.

#### 3.2.1 Participants' experiences of pain and physical symptoms during treatment

Participants' experiences of pain and physical discomfort during anti‐VEGF treatment varied widely, influenced by expectations, somatic sensitivity and situational factors, ranging from minimal discomfort to intense pain.

For some participants, the procedure was less painful than anticipated, leading to a sense of relief and reassurance. Initial anxiety about potential discomfort was replaced by the realization that the injection process was both swift and tolerable: ‘Yes, the treatment itself was no problem… it went so quickly…’ (113). This contrast between expectation and experience often fostered a more positive view of the overall treatment process, and in some cases increased participants' willingness to continue.

However, others reported a more distressing trajectory. While early sessions were described as manageable or even painless, subsequent treatments became increasingly uncomfortable. In these cases, pain was sometimes described as sharp and overwhelming, triggering strong physical and emotional reactions: ‘The first treatment… I didn't feel anything… the second one… (grimaces)… it hurt… and I had this black spot in my eye for over a week… and the last one—it felt like they pushed in a big needle… it hurt so much, terribly’ (104).

Such experiences underscore how pain can escalate unpredictably, creating apprehension about future injections and undermining participants' sense of control.

In contrast, some participants experienced the reverse: initial irritation or pain that gradually diminished over time. As they became more familiar with the procedure, the physical sensations became easier to manage, and in some cases, later treatments were described as entirely painless: ‘… I saw the tip of the needle as he came from the side… and even though it hurt a bit despite the anaesthesia… the last two treatments went really well’ (127).

This adaptation over time may suggest a growing tolerance or increased confidence in the procedure.

For others, the experience of discomfort remained consistent throughout the treatment period. Although unpleasant, the sensations were described as tolerable and did not significantly hinder treatment adherence: ‘It hasn't been a major problem… you feel it when the liquid goes in… like a little flood in the eye… red and sore for a couple of days—nothing you look forward to, but manageable’ (122). These accounts reflect a pragmatic acceptance of mild to moderate side effects as part of the therapeutic process.

Lastly, some participants emphasized the importance of contextual factors, such as the behaviour and technique of the administering physician. Perceptions of pain and comfort were sometimes influenced not just by the injection itself, but by interpersonal dynamics during the procedure: ‘… I thought it went very well… maybe it depends on which doctor—there were three different ones’ (127). These reflections highlight the relational dimension of pain perception, where professional competence and patient‐provider interaction can either mitigate or exacerbate discomfort.

#### 3.2.2 Emotional reactions and concerns

Participants' emotional reactions to anti‐VEGF treatment were varied and shaped by differences in expectations, responses and levels of anxiety.

Some individuals viewed the treatment as a practical necessity rather than an emotionally charged experience. It was simply something that needed to be done: ‘Well, it hasn't really been a big deal… I don't think so’ (122). Such an attitude may have served as a coping strategy aimed at prioritizing the practical demands of treatment while downplaying or avoiding its emotional implications.

For others, the experience was more emotionally charged. Prior to the first injection, feelings of worry, fear and uncertainty were common. In particular, the unknowns regarding the physical and medical aspects of the treatment elicited inner tension: ‘Yes, I was worried before, because I didn't know what kinds of complications there might be or anything like that… but they went through everything so carefully and thoroughly that when I finally had to get the first shot, I felt very calm’ (109). This account illustrates a typical emotional trajectory in which initial anxiety—often linked to uncertainty—was transformed into a sense of calm and trust, particularly when the participant felt well‐informed. It reflects an adaptive process in which emotional distress was mitigated through access to information and a positive relationship with healthcare professionals.

However, it was not only the pain itself that provoked concern. Several participants described a more embodied and intuitive aversion to having someone touch their eye, which is a part of the body perceived as especially private and vulnerable: ‘I wasn't scared, but it was a kind of discomfort… I don't want anyone touching my eyes… it felt so private… I don't quite know how to explain it…’ (120). This reveals a sense of both physical and emotional integrity, where the participant experienced the treatment as an intrusion into their bodily autonomy. This aspect is important to consider, as the preservation of bodily integrity and control can be strongly linked to an individual's overall sense of security and well‐being during medical procedures.

Many participants described a decline in their initial anxiety over time, as the treatment became a routine part of their lives. The previous uncertainty was often replaced by a sense of security and trust in the staff, which helped reduce emotional resistance: ‘I used to feel quite terrified… but the last two times, it's gone really well’ (127). This transformation indicates a successful coping process, in which repeated exposure and the development of trust in the healthcare team reduced initial fears and made the treatment more manageable. Positive treatment experiences—particularly in later stages—contributed to breaking earlier negative emotional patterns, signalling a learned sense of safety and increased tolerance.

Nevertheless, some participants never fully adapted to the treatment. For them, each injection was still accompanied by strong emotional reactions comprising an ongoing mix of anxiety, discomfort and existential uncertainty regarding the treatment's ultimate effectiveness: ‘…before I got these injections, I had undergone laser a few times… and I wonder what happens if I don't respond to these injections. So, I would really like to know—am I facing blindness?’ (132). Here, a deeper existential concern emerges, where the treatment is not only associated with physical discomfort but also with fears about the future, health outcomes and quality of life. In such cases, the treatment becomes more than a clinical intervention; it represents a source of ongoing existential distress.

### 3.3 Uncertainty and variation in visual outcomes

The second category was composed of three subcategories: *participants' experiences of how anti‐VEGF treatment affected their vision*, *uncertainty regarding the long‐term effects of treatment on vision* and *the perceived impact of blood glucose levels on vision*.

#### 3.3.1 Participants' experiences of how anti‐VEGF treatment affected their vision

Participants' experiences of anti‐VEGF treatment effects on vision varied widely, from no improvement to significant functional gains, reflecting individual differences in conditions and needs.

For some participants, the treatment did not result in any noticeable change in visual acuity, leading to a sense of stagnation: ‘I don't notice any difference… no, it's unchanged’ (122). This perception of unchanged vision may reflect a sense of frustration or confusion, where the expectations of improvement were not met. For these individuals, the sense that their vision had not progressed in the desired direction could negatively impact both their motivation and trust in the treatment process.

In other cases, a deeper, existential worry was evident, where participants grappled not only with physical changes but also with uncertainty about their future visual capacity and overall quality of life. These participants described a more disheartening experience, reporting a deterioration in vision despite undergoing treatment. The decline was frequently accompanied by feelings of disappointment and growing concern regarding the long‐term efficacy of anti‐VEGF therapy. One participant expressed the situation as follows: ‘… my vision is worse’ (120). This statement reflected not only a physical decline but also a deeper, existential unease. Participants struggled with the uncertainty surrounding their future visual capacity and the broader implications for their overall quality of life. The emotional burden of declining vision, despite adherence to treatment, underscores the complex interplay between clinical outcomes and participants' well‐being.

Conversely, several participants reported clear improvements in visual function, which were described as both concrete and practical. These improvements extended beyond theoretical enhancements in acuity and included the regained ability to perform daily activities independently: ‘Yes, it has definitely improved… when I go outside, I no longer need my glasses, and I can even read the newspaper now and then’ (104). This reflects a positive re‐evaluation of visual ability, where the patient experienced not only measurable improvement but also a restored sense of independence and control in everyday life.

For some, the effects of treatment varied between sessions. This fluctuation contributed to a sense of unpredictability regarding what to expect from each injection. Some treatments yielded noticeable improvements, while others provided minimal or no perceptible benefit: ‘… the third one made a real difference I thought… after the fourth… I didn't notice any change at all… and the fifth I thought was actually a little better, but it took about a week…’ (217). These episodic treatment experiences suggest a potential sense of uncertainty, as participants could not consistently rely on each injection delivering a predictable or linear improvement. This inconsistency may have contributed to mixed emotional responses and ongoing ambivalence regarding future treatments.

#### 3.3.2 Uncertainty regarding the long‐term effects of treatment on vision

For many participants, treatment raised concerns not only about immediate outcomes but also about the uncertain long‐term effects on vision, especially when improvements were limited or absent.

Some participants expressed relief that their vision had not deteriorated further, but an underlying worry about the future remained. One participant articulated this existential uncertainty and apprehension about what lay ahead: ‘…the only thing you keep thinking about is really… how slow the process might be… if you… so to speak… can avoid any major problems until you die…’ (111). This type of reflection highlights a sense of lost control and existential anxiety regarding a future in which vision may deteriorate regardless of medical efforts. It was not solely the prospect of vision loss that was troubling, but the feeling of being caught in a process that was difficult to predict and even harder to influence.

For others, the uncertainty was even greater, as concerns about the temporary nature of treatment effects were intertwined with fears of future vision loss. There was a more deeply rooted fear that the treatment might not yield lasting results, which in turn gave rise to a sense of inevitable decline. One participant expressed this as follows: ‘…or is it blindness that's waiting?’ (132). This question reflects not only a fear of losing vision but also a fear of losing autonomy and quality of life. These kinds of existential reflections could at times become so overwhelming that they required a deliberate coping strategy to prevent them from dominating everyday life. One participant shared their approach to managing such concerns:

‘…of course I'm worried and I think about it, but… I draw a line so that it doesn't take over…’ (120). This illustrates a conscious effort to maintain a balance between worry and acceptance, establishing boundaries to prevent existential uncertainty from interfering with daily functioning.

#### 3.3.3 The perceived impact of blood glucose levels on vision

Fluctuations in blood sugar levels contributed to unpredictable vision, with high glucose causing blur and more stable levels sometimes improving sight, creating daily practical challenges for participants.

One participant described how their vision was affected by blood sugar in a way that was not only physically noticeable but also emotionally taxing: ‘I get dizzy and nauseous, … I see… things get blurry… Yes, it affects the eyes too, the sugar’ (110). This reflects a clear connection between physical and psychological states, where blood sugar levels influenced not only vision but also emotional and physical well‐being.

While participants were generally aware of the relationship between blood sugar, diet and medication, it was often challenging to maintain stable glucose levels. One participant expressed frustration with the difficulty of managing blood sugar despite understanding its impact on vision: ‘… sometimes I forget to take my diabetic tablet, so then I just take another tablet, you know… that's probably why my blood sugar isn't okay… what worries me is maybe going blind, and then I'm also afraid of a heart attack’ (112). This narrative conveys a sense of helplessness and uncertainty, where the participant struggled to gain or maintain control over both blood sugar and vision, resulting in a constant worry about future complications.

For some, it was also unclear how much of the visual deterioration could be attributed to diabetes and blood sugar levels, further reinforcing the feeling of losing control over one's health. One noted: *‘…sometimes it's the diabetes… Sometimes your levels are too high… and that affects vision too, or? …’* (113). Another said: ‘Yes, when my blood sugar is high, I feel like everything looks blurry… it's not steady… it goes up and down all the time’ (118). These experiences highlight the ongoing uncertainty participants face about whether vision changes are temporary or permanent.

## 4 DISCUSSION

The theme ‘health, hope, and worry’ reflects participants' ambivalent experiences with anti‐VEGF therapy. Hope and worry encompassed emotional and physical challenges during injections, while uncertainty and variation in visual outcomes captured fluctuating perceptions of vision, influenced by factors like blood glucose levels. These findings underscore the need for clear communication, emotional support and trust in healthcare professionals to help people navigate treatment uncertainties.

One of the central themes emerging from the data was the tension between adaptation and distress. While some participants reported becoming accustomed to the regular injections over time, others experienced a cumulative emotional burden, with increasing dread ahead of each visit. This suggests that repeated exposure does not uniformly lead to desensitization or acceptance, contrary to what might be assumed in routine clinical practice. The variability in pain perception further underscores the subjective nature of the treatment experience. For some, pain was minimal or manageable; for others, it remained a central and distressing feature, reinforcing the need for individualized pain management strategies and ongoing assessment.

The emotional vulnerability expressed by many participants was striking. Beyond the physical discomfort, participants described existential concerns about losing their independence, their identity or their ability to participate in meaningful daily activities. These reactions were connected not only to the treatment itself but also to its unpredictable effects. Some reported improved vision, others experienced stagnation, and still others noticed deterioration, despite adhering to the treatment regimen. This unpredictability seemed to undermine a sense of agency and control, contributing to emotional strain and uncertainty about the future. This aligns with previous studies showing that people with diabetes and DME treated with intravitreal injections display considerable variation in psychological responses, fears and expectations.^15^, ^16^

Different psychological profiles have been identified, suggesting that a one‐size‐fits‐all approach may not be effective. A better understanding of these profiles could contribute to improved patient compliance and more tailored support strategies in clinical practice. Many participants also described a fluctuating sense of control related to both their vision and broader diabetes management. The awareness that blood glucose levels could impact eye health created an ongoing pressure to self‐regulate, which in turn evoked feelings of guilt or frustration when vision worsened despite efforts to maintain glycaemic control. This interplay between systemic disease management and ocular outcomes appeared to blur the boundaries of personal responsibility and medical inevitability.

Trust in healthcare professionals emerged as a critical mediator of emotional responses. Participants who felt heard, informed and treated with empathy were more likely to express pragmatic acceptance and adherence to treatment. This is consistent with earlier studies emphasizing the role of the therapeutic relationship in chronic disease management and adherence.^17^, ^18^

These results highlight the need for a holistic approach to anti‐VEGF therapy, where emotional support, continuity of care and attention to patient experiences are integral. Incorporating psychosocial support may improve both well‐being and long‐term treatment adherence.^19^

### 4.1 Methodological considerations

A key strength of this study lies in the fact that the same person conducted the interviews at both time points—approximately 4 months after treatment initiation and again after 1 year. This continuity likely contributed to a greater sense of trust and openness in the interview setting, allowing participants to speak more freely about their experiences.^20^ It also enabled a deeper understanding of individual narratives over time. However, familiarity with participants may also carry a risk of interpretive bias, where the interviewer's prior knowledge of earlier interviews might unintentionally influence the subsequent questioning or interpretation. To mitigate this, a reflective approach was adopted throughout the analysis.^21^

Despite the longitudinal design, capturing the temporal development of participants' experiences was challenging. Some narratives suggested gradual adjustment, while others were fragmented or inconsistent. This may reflect the complexity of experiencing time during ongoing treatment—not as linear, but as a fluid process shaped by vision fluctuations, emotions and daily demands.^22^ The qualitative nature of the study allowed such complexity to be acknowledged rather than simplified.^23^

Another notable feature was the variation in how participants expressed themselves. While some responded in brief or guarded ways, others shared their experiences in greater depth and with rich emotional nuance. This variation may stem from individual communication styles, varying degrees of trust or the sensitivity of the topic. The analysis sought to ensure that all voices were represented equally, so reserved participants were not overshadowed by more verbal ones.

All interviews were carefully transcribed and coded, which facilitated a structured comparison across cases and over time. The coding process allowed the researchers to identify both similarities and differences in how participants described their physical and emotional responses to treatment. By moving between the concrete narratives and more abstract thematic levels, the analysis maintained both closeness to the data and a broader interpretive perspective.^12^

The interview setting and modality (e.g. in‐person or telephone) may also have influenced the depth and character of participants responses. Finally, ethical considerations were central throughout the study. Given the potentially vulnerable position of participants' discussing their eyesight, treatment‐related pain and future uncertainty, particular attention was paid to informed consent, confidentiality and the emotional well‐being of participants' during and after the interviews.

## 5 IMPLICATIONS FOR CLINICAL PRACTICE

These findings suggest that continuity of care and relational continuity may improve treatment adherence and emotional well‐being.

## FUNDING INFORMATION

This study was supported by The Swedish Diabetes Foundation.

## CONFLICT OF INTEREST STATEMENT

The authors declare no conflicts of interest.
